# Childhood undernutrition in North Africa: systematic review and meta-analysis of observational studies

**DOI:** 10.1080/16549716.2023.2240158

**Published:** 2023-07-27

**Authors:** Nagwa Farag Elmighrabi, Catharine A.K. Fleming, Mansi Vijaybhai Dhami, Kingsley E. Agho

**Affiliations:** a School of Health Science, Western Sydney University, Penrith, NSW, Australia; b Department of People Determination and Sustainable Development, Organization of People Determination and Sustainable Development, Benghazi, Libya; c Department of Nutrition, Faculty of Public Health, University of Benghazi, Benghazi, Libya; d Translational Health Research Institute (THRI), Western Sydney University, Penrith, NSW, Australia; e The Children’s Hospital at Westmead Part of the Sydney Children’s Hospitals Network, Sydney, NSW, Australia; f Faculty of Health Sciences, University of Johannesburg, Johannesburg, South Africa

**Keywords:** Stunting, wasting, underweight, determinants, under five years

## Abstract

**Background:**

Undernutrition remains a major public health issue in low- and middle-income countries. Objective Our aim for this study was to identify the factors contributing to undernutrition in children under five years old in North Africa.

**Methods:**

We searched five electronic bibliographic databases (Ovid MEDLINE, Web of Science, Embase (Ovid), ProQuest, and CINAHL) for eligible observational studies published after 2006. STATA version 17 software was used to calculate the odds ratios between associated factors and indicators of undernutrition, with 95% confidence intervals. For each factor, the overall odds were pooled using a forest plot. Due to the significant heterogeneity among the studies (I2 > 50%), a random-effects model was used, and sensitivity analysis was conducted to examine the effect of outliers.

**Results:**

Out of 1093 initially identified studies, 14 met the selection criteria. Our meta-analysis revealed that uneducated mothers were the most common factor associated with undernutrition in North African children. Children aged 0–23 months were significantly associated with stunting (odds ratios (OR) = 1.27; 95% CI: 1.18; 1.37) and wasting (OR = 1.68; 95% CI: 1.42; 1.99). Children living in rural areas were also at higher odds of being stunted (OR = 1.74; 95% CI: 1.64; 1.84) and underweight (OR = 1.59; 95% CI: 1.35; 1.88). These analyses also indicated that a lower wealth index, mothers’ nutritional health, uneducated fathers, and low birth weight were other factors significantly associated with stunting.

**Conclusion:**

Addressing undernutrition in Northern Africa requires a multidisciplinary approach prioritising mothers and young children, especially families in underprivileged areas.

## Introduction

Undernutrition has devastating consequences, leading to a significant increase in mortality and morbidity. Approximately 45% of children annually lose their lives to undernutrition-related causes [1]. Undernutrition heightens the risk and severity of infections and hampers recovery rates by compromising the immune system [2]. The impact of undernutrition on children’s health is far-reaching, with an estimated economic burden of USD 3.5 trillion or USD 500/person annually. In Egypt and Sudan, undernutrition imposes substantial financial costs. In Egypt, the economic burden of undernutrition amounts to approximately EDP 1.1 billion; in Sudan, it is SDG 11.66 billion. Undernutrition is attributed to 11% of child deaths and is projected to rise by 32% by 2025, incurring a cost of around EGP 26.8 billion [3–5]. These alarming statistics highlight the need to address undernutrition and its far-reaching consequences for human lives and economies.

The prevalence of undernutrition (stunting, wasting, and being underweight) poses significant public health challenges to developing countries. Despite ongoing efforts to address the issue, many children continue to suffer. Globally, an alarming 149.2 million children under five years old are affected by stunting, 45 million by wasting, and 13.6 million by severe wasting [6]. This problem is particularly acute in Asia and Africa, where over 90% of children experience undernutrition. The situation is concerning in North Africa’s encompassing countries, such as Egypt, Libya, Sudan, Algeria, Tunisia, Morocco, and the Western Sahara. More than 35% of children under five years old in Libya and Sudan and 22% in Egypt experience stunting [6]. Additionally, over 6.6% of children suffer from wasting despite the region’s previous success in reducing hunger and poverty during the Millennium Development Goals era [6,7]. These statistics highlight the persistent prevalence of undernutrition and its detrimental effects on children’s survival, growth, and development. Intensifying efforts to combat this issue will ensure the well-being and prospects of children in these regions.

Scholars have extensively studied factors influencing children’s nutrition, including age, sex, birth weight, illnesses, caregivers’ health, environmental situations, access to healthcare facilities, rural residence, diet practices, and more [8–17]. These investigations have revealed the complex interplay of determinants contributing to undernutrition in low- and middle-income countries. Additionally, detrimental factors such as poverty, food insecurity, conflicts, climate change, displacement, and infectious conditions significantly worsen the prevalence of undernutrition in these regions [18]. Understanding and addressing these factors is crucial for developing effective strategies to combat undernutrition. The United Nations Children’s Fund (UNICEF) developed a conceptual framework that categorises these factors into basic, underlying, and immediate determinants, providing valuable insights into the interconnected dynamics shaping undernutrition and guiding interventions and policies [19]. Recognising the magnitude and complexity of the problem, the Food and Agricultural Organization (FAO) has underscored the need for collaborative efforts at the national and international levels, involving close monitoring and control of these interconnected components to address undernutrition effectively [20].

High-quality data and evidence in any government development plan are keys to guiding action and monitoring performance [21]. To accelerate progress against undernutrition, investing in research to monitor, assess, and collect up-to-date data, such as national representative data collection surveys and assessment studies, is imperative. This research can help scale cost-effective solutions to improve children’s health and lower undernutrition. Public health planners, policymakers, and implementers can use it to understand the issue and assess the nutritional status of children aged 0–5 to develop initiatives that target communities’ health and well-being. This goal is challenging since North African countries need sub-region-specific information. A recent study assessed the three undernutrition indices among children aged five and under and their determinants in the Eastern Mediterranean (including the countries in this current study) [11]. However, since the scope of this study was limited by the exclusion of Algeria, the results could not be generalised to the entire region. Combined study results from a local area and other areas may be overestimated or underestimated since they are divorced from the local context and must reflect local policies, strategies, or decisions [22].

Thus, we aimed to examine factors contributing to undernutrition in North African children under five years old through a meta-analysis of original research on the topic, specifically in the seven countries mentioned. This study provides information on factors significantly associated with undernutrition in North African children and explains why this region may not achieve Sustainable Development Goals (SDG) Target 2.2 of ‘Ending all forms of malnutrition by 2030’. It also serves as a needs assessment indicator for countries that have reported a high prevalence of undernutrition in children. The review process involved the systematic selection of articles and assessing their validity by synthesising the results qualitatively and quantitatively.

## Methods

The Prospective Registry of Systematic Reviews PROSPERO approved and registered the study protocol under the number (CRD42022324443). Relevant peer-reviewed articles were retrieved by scanning databases, including Ovid MEDLINE, Web of Science, Embase (Ovid), ProQuest, and CINAHL. We identified additional publications through Google Scholar and from the references list sections of articles. Our search context focused on papers from North African countries (Egypt, Sudan, Libya, Algeria, Morocco, Tunisia, and Western Sahara) published between 1 January 2006 and 15 May 2022. In this review, 2006 served as a baseline because the World Health Organisation (WHO) introduced its Child Growth Standards that year [23]. We used the following terms in our search process across five databases:

(Child* or under-five* or paediatr* or infant* or bab*) and (Malnutr* or malnourish* or undernourish* or undernutr* or stunt* or wast* or underweight*) and (Egypt* or Sudan* or Libya* or Algeria* or Tunisia* or Morocco* or Western Sahara* or North Africa*) and (factor* or determinant* or correlate* or cause*).

### Eligibility criteria

We conducted an eligibility assessment for this review and only included studies that (i) focused on undernutrition in children under five years old; (ii) were conducted in North Africa; (iii) reported factors associated with undernutrition; (iv) were published between 1 January 2006 and 15 May 2022; (v) were observational studies (cross-sectional studies, cohort studies, and case–control studies); (vi) were published in a peer-reviewed journal (non-peer-reviewed research, reviews, commentaries, letters to editors, and conference presentations were excluded); and (v) were written in English. The decision to include all North African countries (Egypt, Sudan, Libya, Algeria, Tunisia, Morocco, and Western Sahara) was based on a previous study conducted by Elmighrabi et al. (2023).

### Data extraction

We uploaded the articles selected from each database into EndNote X20 (Clarivate Analytics, USA) to arrange, organise, stimulate, and export the referenced bibliographies in a scientific and systematic way [24]. After removing duplicates, the first author, N.F.E., reviewed the study titles and screened all the publications. Following the initial screening, the second phase involved reading abstracts and retaining eligible studies. The third and final screening involved reading the full text and retaining studies that met the eligibility.

### Reliability

A second reviewer (M.V.D.), blinded to the primary reviewer (N.F.E.), assessed the article selection and data extraction processes. At each stage, we checked the number of articles and discussed any differences of opinion. A third reviewer (K.E.A.) was available to clarify and confirm the final publications identified for inclusion.

### Quality assessment

We used the National Heart, Lung, and Blood Institutes (NIH) tool to evaluate the quality of each paper [25]. Each reviewed study was scored, with a possible range of 0 to 14 points (0 if no criteria were met and 14 if all criteria were met). The sum of the points determined the overall quality of the paper. Studies were rated as being high quality (≥10), medium (6–9), and poor quality (≤5). A study with a low-quality rating was more likely to be biased and vice versa. Table S2 includes the quality assessment questions and assigned scores for each paper.

### Meta-analysis

Since these study analyses were heterogeneous, our analysis was divided into three sections: a systematic qualitative review, a meta-analysis of factors investigated in two studies or more, and an assessment of factors reported in only one study. Studies identifying associated factors based on odds ratios with a 95% confidence interval were entered into the system for analysis. We manually calculated odds ratios and confidence intervals (CIs) in studies that reported coefficients. All undernutrition-related factors that met the eligibility criteria of two or more studies were identified, managed, and exported to STATA 17 for further analysis. Subgroup analyses were conducted if needed for each factor or category, as per children’s age. We used the funnel plot to display biases and study effects, and we evaluated asymmetry using Begg’s test. The forest plot for undernutrition, including stunting, wasting, and underweight, is shown in Figures S1-S3. Each point on the plot corresponds to the log odds ratio from each study, and the dashed lines represent the expected 95% confidence intervals around the summary estimate.

## Results

**Figure 1.**
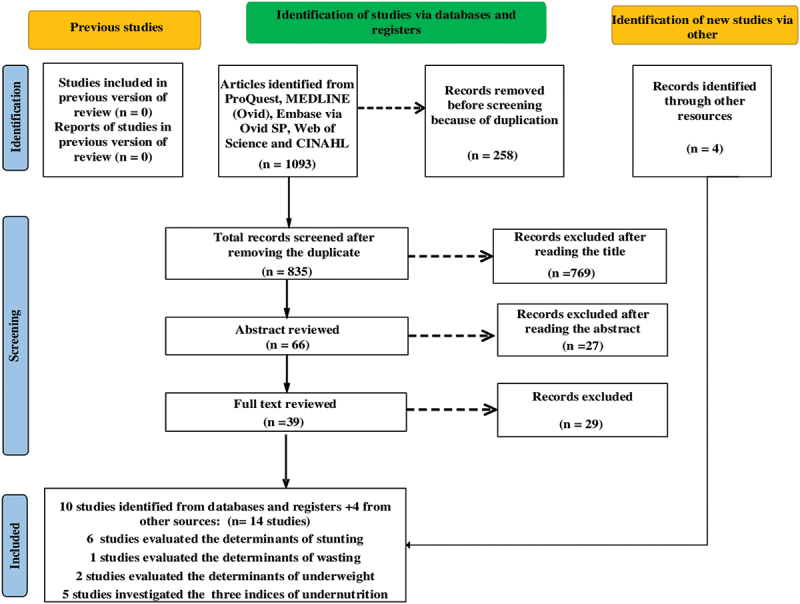
The PRISMA 2020 flow diagram of a systematic review and meta-analysis of the factors associated with undernutrition in North Africa.

According to Figure 1, a total of 1,093 studies were found across the five databases. After removing 258 duplicates, there were 835 studies left to screen. An evaluation of the titles eliminated 769 studies, as they did not satisfy the eligibility criteria. We eliminated another 27 studies after surveying the abstracts of the remaining 66 as they did not indicate undernutrition-related determinants or factors. We examined the full texts of the remaining 39 studies and eliminated 29 studies as they did not meet one or more criteria. Of the original 1093 studies identified, 10 met the inclusion criteria. We identified four additional studies from outside sources. Accordingly, 14 studies [26–39] were identified for critical review.

### Characteristics of included studies

**Figure 2.**
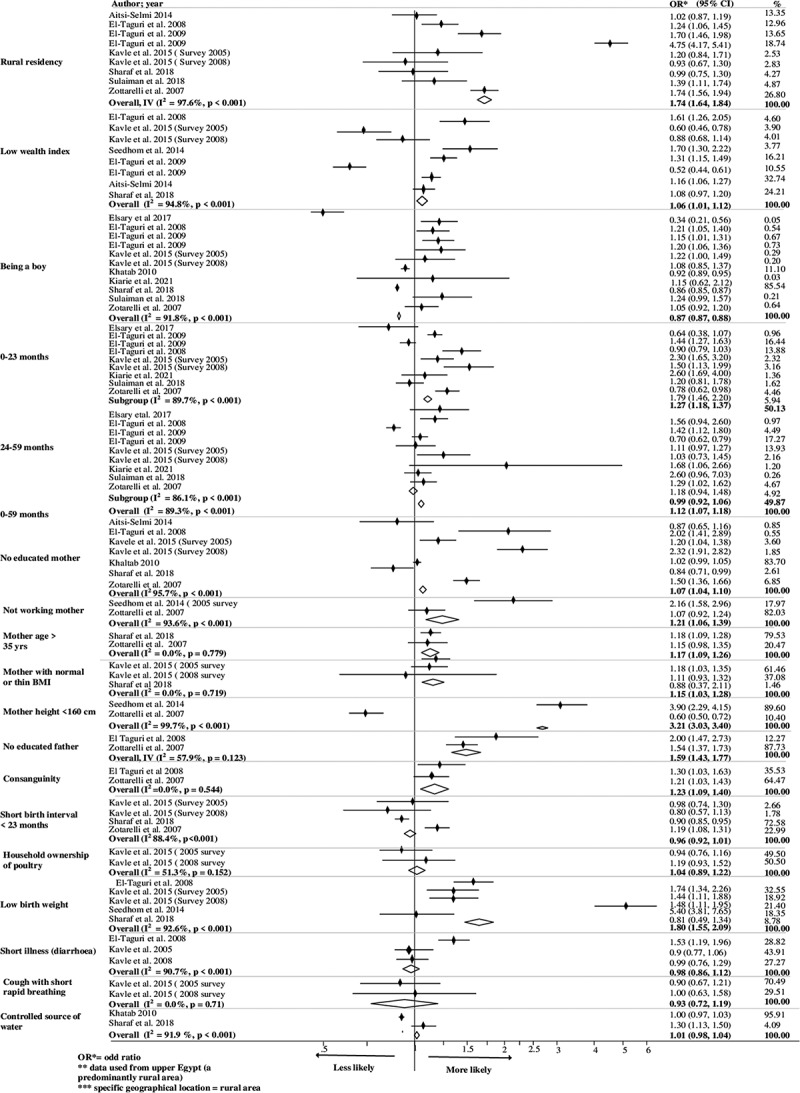
Factors associated with undernutrition (stunting) among children under five in North Africa.

**Figure 3.**
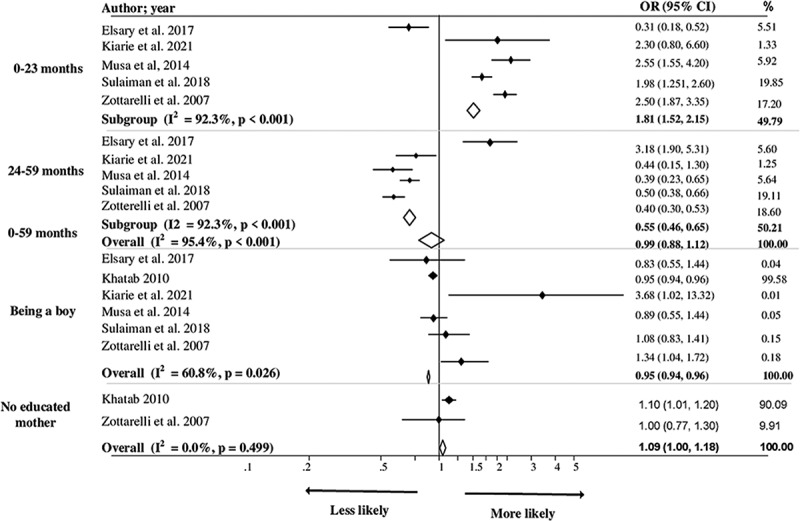
Factors associated with undernutrition (wasting) among children under five in North Africa.

**Figure 4.**
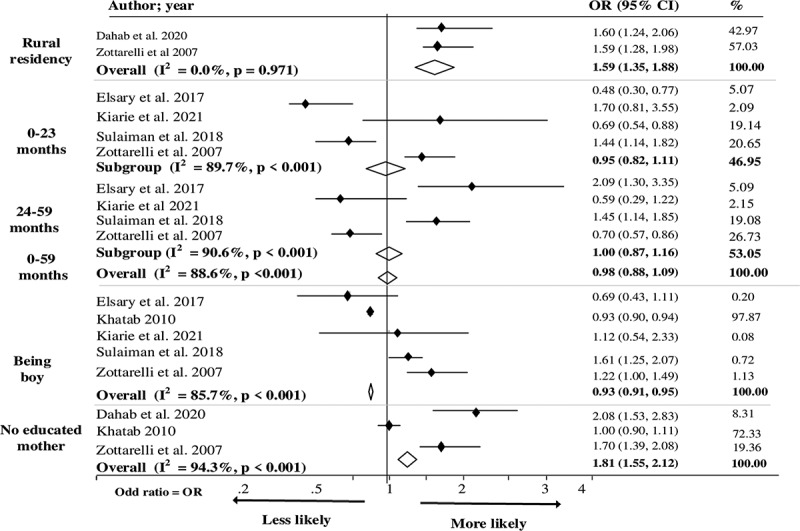
Factors associated with undernutrition (underweight) among children under five in North Africa.

Table 1 shows 14 studies categorised by the anthropometric indices of undernutrition (stunting, wasting, and being underweight), which comprise 13 cross-sectional studies and 1 matched case–control study. Eight of the identified studies were conducted in Egypt [26,28,29,32–34,38,39], four in Sudan [27,30,31,37], and two in Libya [35,36], with one of the latter two conducted in both Libya and Morocco [36]. The study incorporated 84,997 children aged 0–5. Only one case–control study was included [39], and the rest were cross-sectional studies [26–38]. Thirteen of the 14 studies were analysed, including 84,797 children aged 0–5 [26–38]. We excluded the case–control study from the analysis to avoid affecting the study results [38]. Eight studies focused on children aged 0–5 years old [26,27,30,32,34–37]. Among the studies identified, six focused on stunting [28,29,33,35,36,38], one on wasting [30], two on being underweight [37,39], and five examined all three indices of undernutrition in the region [26,27,31,32,34]. The 14 criteria used to measure the study quality revealed that 93% were of medium quality and 7% were of high quality, as shown in S2 Table. We used a forest plot and pooled analysis to determine association significance when factors were investigated by two or more studies (Figures 2–4).

**Table 1.** Characteristics of included publications in the literature.

No	Author; year	Region	Type and year of survey	Children (N)	age	Study design	Residence	Statistical measure of association	Measured index
1	AbdElAziz and Hegazy 2012	Egypt	2010	N=200	6–24 months	Case control study	Rural-urban	odds ratio and *P* value	Underweight
2	Aitsi-Selmi 2014	Egypt	2008	N= 5357	0–3 years	Cross-sectional study	Rural-urban	Proportion	Stunting
3	Dahab et al., 2020	Sudan	2014	N= 14081	0–5 years	Cross-sectional study	Rural-urban	Proportion and odds ratio	Underweight
4	Elsary et al. 2017	Egypt	2014	N=400	0–5 years	Cross-sectional study	Rural	Proportion and *P* value	Stunting, wasting and underweight
5	El Taguri et al., 2008	Libya	1995	N= 4549	0–5 years	Cross-sectional study	Rural-urban	odd ratio and *P* value	Stunting
6	El Taguri et al. 2009	Libya	2003	N = 7232	0–5 years	Cross-sectional study	Rural-urban	Proportion, risk ratio, and confidence interval	Stunting
		Morocco	2003	N= 5380	0–5 years		Rural-urban		
7	Kavle et al., 2015	Egypt	2005	N= 6091	6–59 months	Cross-sectional study	Rural-urban	Proportion and odds ratio	Stunting
			2008	N= 7794	6–59 months		Rural-urban		
8	Khatab 2010	Egypt	2003	N= 6661	0–5 years	Cross-sectional study	Rural-urban	Geoadditive Gaussian and latent variable models	Stunting, wasting and underweight
9	Kiarie et al., 2021	Sudan	2018	N= 630	6–59 months	Cross-sectional study	Rural	Odds ratio and proportion and *P* value	Stunting, wasting and underweight
10	Musa et al. 2014	Sudan	2014	N= 411	0–5 years	Cross-sectional study	Urban	Proportion and confidence intervals	Wasting
11	Seedhom et al. 2014	Egypt	2014	N=700	6–24 months	Cross-sectional study	Rural	Proportion, odds ratio, and *P* value	Stunting
12	Sharaf et al. 2018	Egypt	2014	N= 13682	0–4 years	Cross-sectional study	Rural-urban	Three-fold decomposition	Stunting
13	Sulaiman et al. 2018	Sudan	2014	N=1635	0–5 years	Cross-sectional study	Rural	Proportion and confidence intervals	Stunting, wasting and underweight
14	Zottarelli et al. 2007	Egypt	2000	N=10194	0–5 years	Cross-sectional study	Rural-urban	Odds ratio, standard error, and *P* value	Stunting, wasting and underweight

Abdelaziz and Hegazy [39] conducted a matched case–control study in Cairo, Egypt, and found that women who were unemployed, illiterate, and did not visit health or nutrition clinics were associated with undernutrition. Undernutrition was significantly higher among women who did not exclusively breastfeed or delayed breastfeeding.

### Factors associated with stunting

Figure 2 illustrates several factors associated with stunting in North Africa. Statistically, rural children had a higher risk of stunting, with an overall pooled odds of 1.74 and a 95% confidence interval (CI) of (1.64, 1.84). Khattab [32] observed that children in urban areas were protected from stunting. El Taguri [35] also observed the significant impact of environmental factors such as dwelling type, kitchen location, water storage, toilet facility, and garbage disposal methods on stunting among children under five years old. Both Sulaiman et al. [27] and El Taguri [35] found that stunting was prevalent in specific locations, such as the Berber region of Sudan or the Al-Akhdar district in Libya. In addition, a positive association was found between stunting and families with a lower wealth index, with a pooled odds ratio of 1.06 and 95% CI of (1.01, 1.12).

We also explored the impact of a child’s age, birth weight, and health on the presence of stunting. We found that stunting was more prevalent in younger children and in children who had a low birth weight [Overall pooled OR: 1.27; 95% CI = (1.18, 1.37)] and [Overall pooled OR: 1.80; 95% CI = (1.55, 2.09)], respectively. Boys were less likely to be stunted [Overall pooled OR: 0.87; 95% CI = (0.87, 0.88)]. Although we did not find a statistical association between childhood, illnesses (diarrhoea) and stunting, Elsary et al. [34] found an important association between illness (gastroenteritis and parasitic infestation) and stunting among Egyptian children under five years old. See Table S4 for details.

Our results also showed the significant effects of socio-demographic factors on children’s nutrition. Stunting was more likely among children with uneducated mothers [Overall pooled odds ratio (OR): 1.07; 95% (CI) = (1.04, 1.10)], unemployed mothers [Overall pooled OR: 1.21; 95% CI = (1.06, 1.39)], and uneducated fathers [Overall pooled OR: 1.59; 95% CI = (1.43, 1.77)]. Statistically, stunting was also positively associated with a mother’s height <160 cm [Overall pooled OR: 3.21; 95% CI = (3.03, 3.40)] and normal or low Body Mass Index (BMI) [Overall pooled OR: 1.15; 95% CI = (1.03, 1.28)]. Blood-related parents had a higher chance of bearing stunted children [Overall pooled OR: 1.23; 95% CI = (1.09, 1.40)]. Antenatal care was statistically associated with stunting with a pooled odds ratio of 1.03 and 95% CI of (1.00, 1.06). Among North African children under five years old, stunting was also associated with the number of children <5 years old in a family, the birth order of children > 5, being uninsured, watching television, a lack of playtime with family, lack of visits reliance on agriculture, mother’s nutritional knowledge, early weaning, frequent complementary feeding (four to six times), and introducing complementary feeding at an early age [26,28,29,34,35]. We used funnel plots and Begg’s test for stunting and found that the estimated bias coefficient was 3.56 with a standard error of 0.62 (*P* < 0.001), indicating strong evidence for the presence of small-study effects (Figure S1).

### Factors associated with wasting

Younger children (children aged 0–23 months) and children with uneducated mothers were at the highest risk of wasting among children under five in the North African region [Overall pooled OR: 1.68; 95% CI = (1.42, 1.99)] and [Overall pooled OR: 1.09; 95% CI = (1.00, 1.18)], respectively (see Figure 3).

Considering the factors evaluated in one study, Sulaiman et al. [27] concluded that Atbara had the highest prevalence of wasting. Kiarie et al. [31] found that non-residential status was significantly associated with wasting among children in Sudan. Elsary et al. [34] identified an association between wasting and improper weaning, gastroenteritis, and parasitic infestation factors in Egyptian children (Table S4). Figure S2 presents a funnel plot and Begg’s test, which revealed some evidence for the presence of small-study effects (P = 0.517).

### Factors associated with being underweight

No significant association was found between age and being underweight in North African children, which differs from our previous findings regarding stunting and wasting. Figure 4 shows that the risk of being underweight was highest among children living in rural areas [Overall pooled OR: 1.59; 95% CI = (1.35, 1.88)] and children with uneducated mothers [OR: 1.81; 95% CI = (1.55, 2.12)]. Sulaiman et al. [27] recognised that living in a specific locality (Abo Hamad) was significantly associated with underweight children under five years old. Zottarelli et al. [26] and Elsary et al. [34] found that maternal height, maternal age >35 years old, uneducated fathers, consanguinity (first degree), child order > 5, childbirth interval <23 months, and childhood illnesses (gastroenteritis and parasitic infestation) were strongly associated with underweight Egyptian children under five years old. Kiarie et al. [31] and Dahab et al. [37] also found that family size of 9 people ± 4, poor household wealth index, and diarrhoea were associated with being underweight in Sudanese children under five years old (see Table S4). Figure S3 suggests evidence for small-study effects based on a funnel plot and Begg’s test with an estimated bias coefficient of 1.87 (*P* = 0.032). A meta-regression analysis on undernutrition, based on the year of publication, showed a decrease in undernutrition for every one-unit increase in the log odds (Figure S4).

## Discussion

Our critical review of 14 North African studies highlights key factors linked to undernutrition (stunting, wasting, and being underweight) among children under five years old. Among the most common factors identified were uneducated mothers, young children (<23 months), and living in rural areas. Additional factors found in our pooled analysis included lower household wealth, uneducated fathers, unemployed mothers, older maternal age, parental blood relationship, low birth weight, and certain feeding-related factors. Among North African children, undernutrition was less likely to occur in boys, households with controlled water, and the absence of childhood illnesses.

Our results were consistent with several studies on some points, but not with others. For example, our results were consistent with Akombi et al. [10] in Sub-Saharan Africa, Al-Shameri et al. [11] in the Middle East and North African countries, and Chopra [13] in South Africa, who found a strong association between undernutrition and low socioeconomic status, living in rural areas, the environment, and maternal nutritional health. However, our study explored a considerable connection between undernutrition at younger ages. We found that undernutrition was less likely among boys, which does not agree with earlier studies [10,11,13,16,40–42]. The first two years of a child’s life are critical to their well-being and development. Insufficient nutrition during this crucial phase can lead to severe weight loss and impair the child’s growth [42]. Similarly, during pregnancy, maternal health and nutrition are of the utmost importance and must remain so for the first six months post-birth, when the infant wholly relies on their mother for sustenance [42–44]. Improper nourishment during pregnancy and the first two years of a child’s life can lead to widespread undernutrition, especially in developing nations, including North Africa.

Our analysis highlights the crucial role of timing and method in introducing solid foods to North African children, which affect their undernutrition status. We found that factors such as sudden or improper weaning, inappropriate complementary feeding practices, and the timing and frequency of introducing and discontinuing exclusive breastfeeding were significant contributors. It is essential that mothers exclusively breastfeed their child for the first six months to prevent growth deficits [43]. However, after six months, introducing adequate complementary foods is necessary for optimal growth. The periods between 0–6 months, when breastfeeding is the sole source of nutrition, and 6–23 months, when new foods are introduced, represent crucial stages where children are highly vulnerable to undernutrition [45,46]. Prolonged breastfeeding without appropriate supplementation with high-quality, sufficient, and frequent complementary foods can lead to undernutrition and recurrent illness [46].

Our meta-analysis findings indicate a significantly higher likelihood of stunted and underweight children under five years old in rural areas than in urban areas. The undernutrition observed in rural areas can be attributed to several factors: 1) Limited access and lower rates of timely prenatal care among rural mothers, often leading to home births compared to urban areas. 2) Inadequate provision of preventative medical and dental care as well as recommended vaccinations for infants and toddlers in rural areas. 3) Rural families with lower incomes often have fewer health insurance coverage options than urban families [47]. Furthermore, specific rural locations exhibit a higher prevalence of undernutrition in children under five years old potentially due to marginalisation, which can worsen environmental conditions and children’s health [9,13]. Our review underscores the significant association between children’s undernutrition in North Africa and environmental conditions based on water source, dwelling type, toilet facility, and garbage disposal [35]. These unfavourable environmental conditions are evident in rural areas and underprivileged urban regions [9,27,36].

Undernutrition is not confined to rural areas alone, even though children from rural or specific areas are more susceptible to undernutrition compared to their urban counterparts. Our meta-analysis revealed that stunting was prevalent among families with lower wealth indexes and caregivers facing various related conditions, regardless of living in a rural or urban area. The prevalence of stunting and being underweight was higher in families with uneducated parents and/or unemployed mothers, significantly affecting the family’s income, limited access to health services, and limited ability to purchase food. These conditions can affect the health and nutrition of both mother and child and increase their risk of disease and/or growth deficiencies [3,9–13].

Furthermore, our research revealed that North African children from large-family size either in terms of the number of family members or higher birth order, had lower nutrition indices. This finding reflects research results in other parts of the world [10,48]. Households with more members tend to have lower childcare and dietary intake levels, particularly those with lower incomes [49]. Large families living on a low income can exacerbate undernutrition in children under five years old, particularly in communities with fewer or no social security services for disadvantaged families. These results were consistent with many previous studies [50–52].

The review highlights that the three indices of undernutrition, including stunting, were significantly associated with gastroenteritis, parasitic infestation, and diarrhoea. The double disadvantage of inadequate dietary intake and an unhealthy environment is increasing children’s susceptibility to illnesses such as diarrhoea, infection, and fever. In turn, illness suppresses the child’s appetite, inhibits the absorption of nutrients from food, and increases their need for caloric availability [53]. These results were consistent with a meta-analysis of undernutrition prevalence and associated factors among primary school-aged children in Ethiopia [54].

Our results revealed that a child’s birth weight affects their nutrition and health; it was also significantly associated with stunting in our meta-analysis. Children with low birth weight are susceptible to infections such as diarrhoeal and lower respiratory infections [15,55], which contribute to undernutrition. Low birth weight is also associated with poor maternal nutrition, low maternal BMI, height <160 cm, low educational levels, and lack of access to antenatal care [12]. These factors arguably clarify the significant association between the pooled odds ratio of normal and low maternal BMI and height <160 cm with stunting in North African children.

This review stresses the need to improve the nutritional status of North African children through the following goals: Firstly, improve education and employment opportunities in disadvantaged rural areas since higher education and income levels can improve wider environmental conditions and promote stronger health and nutrition outcomes. Secondly, introduce parental programmes about the importance of nutrition and health, family planning, healthcare, breastfeeding, and complementary feeding, particularly in rural areas with low socioeconomic status. Raising awareness and knowledge about health and nutrition can help improve the health of children, parents, and the wider community in the short and long terms. Thirdly, ensure that primary health care services are available and accessible, especially in disadvantaged areas. Improving the infrastructure and environment, e.g. increasing access to safe water supplies, establishing sanitary facilities, and providing safe housing, is essential to preventing the spread of illness among children under five years old, especially in rural or disadvantaged areas. Our study findings can help governments, policymakers, public health researchers, non-government aid organisations, and other stakeholders address undernutrition causes in North Africa. This information can also help them proactively combat the burden of undernutrition and allocate resources to North African countries including Egypt, Sudan, Libya, Algeria, Morocco, Tunisia, and the Western Sahara.

### Study strengths and limitations

The strengths of our study include its comprehensive and systematic search of five databases to identify relevant and recent articles on children’s undernutrition and its associated factors in North African countries. Strict adherence to PRISMA during the review process ensured rigour. Furthermore, using the WHO’s 2006 standards for children’s health created a standard baseline for the articles. In combination, statistical analysis was used to summarise and quantify the results from individual studies and the three-party review process promoted the findings’ reliability. However, this review has some limitations. Almost all the identified studies were cross-sectional, which might affect the temporal relationship between undernutrition factors and outcomes of concern for children under five years old in North African countries. Using PRISMA, we determined that all eligible studies were biased to an acceptable level. Selecting English-only studies biases the results towards predominantly English-speaking countries, thus limiting the potential impact and dissemination of this review. Other limitations associated with this current study include: 1) cohort studies were not included in our review despite possessing strengths for establishing causality and controlling confounding factors. This lack was primarily due to the limited availability or absence of relevant cohort studies on the topic of interest. Consequently, surveys were utilised as the primary source of evidence, offering valuable insights into the prevalence, associations, and patterns of variables under investigation. 2) Although our search strategy aimed to encompass all seven North African countries, we could only obtain research specific to Egypt, Libya, Morocco, and Sudan. 3) Additionally, it is worth noting that converting the beta coefficients to odds ratios could potentially lead to overestimation or underestimation of the odds ratios.

## Conclusion

The study illustrates the multifaceted factors contributing to undernutrition in North African children. Strategies at various levels must be implemented to address this issue effectively. Governments and other stakeholders in North Africa should focus on establishing health and nutrition initiatives targeting mothers and young children, especially families from disadvantaged areas. Parental education programmes, immunisation programmes, and family planning can significantly improve maternal and child nutrition and health. These initiatives should be launched promptly and sustained in the long term to positively impact undernutrition and health outcomes in North African children, mothers, and the broader population.

## Supplementary Material

Supplemental Material

## Paper context

During the Millennium Development Goals era, North Africa made significant strides in reducing poverty and hunger. However, the region faces a worsening undernutrition problem, indicating potential challenges in achieving Sustainable Development Goals Target 2.2 by 2030, which aims to eradicate all forms of malnutrition. We identified various factors contributing to undernutrition in North African children through our meta-analysis and systematic review. Our findings highlight the urgency to implement multi-level strategies targeted at disadvantaged families.
