# Supplementary material for: Childhood undernutrition in North Africa: systematic review and meta-analysis of observational studies
Source: Glob Health Action. 2023 Jul 27;16(1):2240158. doi: 10.1080/16549716.2023.2240158 (PMC10375933; doi:10.1080/16549716.2023.2240158)
Supplement: Supplemental Material [file ZGHA_A_2240158_SM4303.zip › Suplementary_Figures.docx]

**Figure S1.** Funnel plots and 95% Confidence Intervals (CIs) of stunting.

**Figure S2.** Funnel plots and 95% Confidence Intervals (CIs) of wasting.

**Figure S3.** Funnel plots and 95% Confidence Intervals (CIs) of being underweight.

Fig S1-S3 indicated the forest plot for the undernutrition (stunting wasting and underweight) The points correspond to the log odds ratio from each study, and the dashed lines show the expected 95% confidence intervals around the summary estimate.

**Figure S4.** A meta-regression analysis of undernutrition by year of publication.

The vertical axis is the log proportion of undernutrition, and the horizontal axis represents the year of publications. Each red dot represented one selected study, and the size of each red dot corresponds to the weight assigned to each study. Given that the slope of the regression line has descended slightly in this figure, this could be interpreted as the publication of the year indicating a weak strong association with the proportion of undernutrition. For every one-unit increase in undernutrition decreases the publication year and but this association differs statistically (P < 0.001)
