# Supplementary material for: Childhood undernutrition in North Africa: systematic review and meta-analysis of observational studies
Source: Glob Health Action. 2023 Jul 27;16(1):2240158. doi: 10.1080/16549716.2023.2240158 (PMC10375933; doi:10.1080/16549716.2023.2240158)
Supplement: Supplemental Material [file ZGHA_A_2240158_SM4303.zip › Suplementary_Tables.docx]

**Table S1. PRISMA 2020 checklist for the main.**

|  | **Item #** | **Checklist item** | **Location where the item is reported** |  |  |
| --- | --- | --- | --- | --- | --- |
| **TITLE** | | |  |  |  |
| Title | **1** | Identify the report as a systematic review. | Title |  |  |
| **ABSTRACT** | | |  |  |  |
| Abstract | **2** | See the PRISMA 2020 for Abstracts checklist. | Table S2 |  |  |
| **INTRODUCTION** | | |  |  |  |
| Rationale | **3** | Describe the rationale for the review in the context of existing knowledge. | Introduction |  |  |
| Objectives | **4** | Provide an explicit statement of the objective(s) or question(s) the review addresses. | Introduction |  |  |
| **METHODS** | | |  |  |  |
| Eligibility criteria | **5** | Specify the inclusion and exclusion criteria for the review and how studies were grouped for the syntheses. | Methods |  |  |
| Information sources | **6** | Specify all databases, registers, websites, organisations, reference lists and other sources searched or consulted to identify studies. Specify the date when each source was last searched or consulted. | Methods |  |  |
| Search strategy | **7** | Present the full search strategies for all databases, registers and websites, including any filters and limits used. | Methods |  |  |
| Selection process | **8** | Specify the methods used to decide whether a study met the inclusion criteria of the review, including how many reviewers screened each record and each report retrieved, whether they worked independently, and if applicable, details of automation tools used in the process. | Methods |  |  |
| Data collection process | **9** | Specify the methods used to collect data from reports, including how many reviewers collected data from each report, whether they worked independently, any processes for obtaining or confirming data from study investigators, and if applicable, details of automation tools used in the process. | Methods |  |  |
| Data items | **10a** | List and define all outcomes for which data were sought. Specify whether all results that were compatible with each outcome domain in each study were sought (e.g. for all measures, time points, analyses), and if not, the methods used to decide which results to collect. | Methods |  |  |
|  | **10b** | List and define all other variables for which data were sought (e.g. participant and intervention characteristics, funding sources). Describe any assumptions made about any missing or unclear information. | Methods |  |  |
| Study risk of bias assessment | **11** | Specify the methods used to assess risk of bias in the included studies, including details of the tool(s) used, how many reviewers assessed each study and whether they worked independently, and if applicable, details of automation tools used in the process. | Methods |  |  |
| Effect measures | **12** | Specify for each outcome the effect measure(s) (e.g. risk ratio, mean difference) used in the synthesis or presentation of results. | Methods |  |  |
| Synthesis methods | **13a** | Describe the processes used to decide which studies were eligible for each synthesis (e.g. tabulating the study intervention characteristics and comparing against the planned groups for each synthesis (item #5)). | Methods |  |  |
|  | **13b** | Describe any methods required to prepare the data for presentation or synthesis, such as handling of missing summary statistics, or data conversions. | Methods |  |  |
|  | **13c** | Describe any methods used to tabulate or visually display results of individual studies and syntheses. | Methods |  |  |
|  | **13d** | Describe any methods used to synthesize results and provide a rationale for the choice(s). If meta-analysis was performed, describe the model(s), method(s) to identify the presence and extent of statistical heterogeneity, and software package(s) used. | Methods |  |  |
|  | **13e** | Describe any methods used to explore possible causes of heterogeneity among study results (e.g. subgroup analysis, meta-regression). | Methods |  |  |
|  | **13f** | Describe any sensitivity analyses conducted to assess robustness of the synthesized results. | Methods |  |  |
| Reporting bias assessment | **14** | Describe any methods used to assess risk of bias due to missing results in a synthesis (arising from reporting biases). | Methods |  |  |
| Certainty assessment | **15** | Describe any methods used to assess certainty (or confidence) in the body of evidence for an outcome. | Methods |  |  |
| **RESULTS** | | |  |  |  |
| Study selection | **16a** | Describe the results of the search and selection process, from the number of records identified in the search to the number of studies included in the review, ideally using a flow diagram. | Figure 1  flow chart |  |  |
|  | **16b** | Cite studies that might appear to meet the inclusion criteria, but which were excluded, and explain why they were excluded. | Methods |  |  |
| Study characteristics | **17** | Cite each included study and present its characteristics. | Methods |  |  |
| Risk of bias in studies | **18** | Present assessments of risk of bias for each included study. | Results |  |  |
| Results of individual studies | **19** | For all outcomes, present, for each study: (a) summary statistics for each group (where appropriate) and (b) an effect estimates and its precision (e.g. confidence/credible interval), ideally using structured tables or plots. | Results |  |  |
| Results of syntheses | **20a** | For each synthesis, briefly summarise the characteristics and risk of bias among contributing studies. | Results |  |  |
|  | **20b** | Present results of all statistical syntheses conducted. If meta-analysis was done, present for each the summary estimate and its precision (e.g. confidence/credible interval) and measures of statistical heterogeneity. If comparing groups, describe the direction of the effect. | Results |  |  |
|  | **20c** | Present results of all investigations of possible causes of heterogeneity among study results. | Results |  |  |
|  | **20d** | Present results of all sensitivity analyses conducted to assess the robustness of the synthesized results. | Results |  |  |
| Reporting biases | **21** | Present assessments of risk of bias due to missing results (arising from reporting biases) for each synthesis assessed. | Results |  |  |
| Certainty of evidence | **22** | Present assessments of certainty (or confidence) in the body of evidence for each outcome assessed. | Results |  |  |
| **DISCUSSION** | | |  |  |  |
| Discussion | **23a** | Provide a general interpretation of the results in the context of other evidence. | Discussion |  |  |
|  | **23b** | Discuss any limitations of the evidence included in the review. | Discussion |  |  |
|  | **23c** | Discuss any limitations of the review processes used. | Discussion |  |  |
|  | **23d** | Discuss implications of the results for practice, policy, and future research. | Discussion |  |  |
| **OTHER INFORMATION** | | |  |  |  |
| Registration and protocol | **24a** | Provide registration information for the review, including register name and registration number, or state that the review was not registered. | Registration and protocol |  |  |
|  | **24b** | Indicate where the review protocol can be accessed, or state that a protocol was not prepared. | Registration and protocol |  |  |
|  | **24c** | Describe and explain any amendments to information provided at registration or in the protocol. | Registration and protocol |  |  |
| Support | **25** | Describe sources of financial or non-financial support for the review, and the role of the funders or sponsors in the review. | Funding |  |  |
| Competing interests | **26** | Declare any competing interests of review authors. | Conflict of interest |  |  |
| Availability of data, code, and other materials | **27** | Report which of the following are publicly available and where they can be found template data collection forms; data extracted from included studies; data used for all analyses; analytic code; any other materials used in the review. | Availability of data and other materials |  |  |

**Table S2.** Quality assessment score.

| **Author; year** | **1- Was the**  **research**  **question or**  **objective in this paper**  **clearly**  **stated?** | **2- Was the study**  **population**  **clearly**  **specified and**  **defined?** | **3-Was the**  **participation**  **rate of eligible persons at least 50%?** | **4- Were all the subjects**  **selected or recruited**  **from the same or similar populations**  **(Including the same**  **time)? Were**  **inclusion and**  **exclusion criteria for**  **being in the study**  **prespecified and applied uniformly to**  **all participants?** | **5- Was a sample**  **size**  **justification,**  **power**  **description, or variance and effect estimates**  **provided?** | **6- For the analyses in this paper, were the exposure(s) of interest measured prior to the outcome(s) being measured?** | **7- Was the timeframe sufficient so that one could reasonably expect to see an association between exposure and outcome if it existed?** | **8- did the study examine different levels of the exposure as related to the outcome (e.g., categories of exposure, or exposure measured as continuous variable)?** | **9- Were the exposure measures (independent variables) clearly defined, valid, reliable, and implemented consistently across all study participants?** | **10- Was the**  **exposure(s)**  **assessed more**  **than once over**  **time?** | **11- Were the**  **exposure**  **measures**  **(Dependent**  **variables)**  **clearly defined,**  **valid, reliable,**  **and**  **implemented**  **consistently**  **across all study**  **participants?** | **12- Were the**  **outcome**  **assessors**  **blinded to the**  **exposure status**  **of participants?** | **13- Was loss to follow‐up after baseline 20% or less?** | **14- Were key**  **potential**  **confounding**  **variables**  **measured and**  **adjusted**  **statistically for**  **their impact on**  **the relationship**  **between**  **exposure(s) and**  **outcome(s)?** | **Quality**  **score** |
| --- | --- | --- | --- | --- | --- | --- | --- | --- | --- | --- | --- | --- | --- | --- | --- |
| **AbdElAziz and**  **Hegazy 2012** | 1 | 1 | 1 | 1 | 0 | 0 | 0 | 1 | 1 | 0 | 1 | 0 | 1 | 1 | 9 |
| **Aitsi-Selmi 2014** | 1 | 1 | 1 | 0 | 1 | 0 | 0 | 1 | 1 | 1 | 1 | 0 | 1 | 0 | 9 |
| **Dahab et al 2020** | 1 | 1 | 1 | 1 | 1 | 0 | 0 | 1 | 1 | 0 | 1 | 0 | 1 | 0 | 9 |
| **Elsary et al 2017** | 1 | 1 | 1 | 1 | 0 | 0 | 0 | 1 | 1 | 0 | 1 | 0 | 1 | 0 | 8 |
| **El Taguri et al. 2008** | 1 | 1 | 1 | 0 | 1 | 0 | 0 | 1 | 1 | 0 | 1 | 0 | 1 | 0 | 8 |
| **El Taguri et al. 2009** | 1 | 1 | 1 | 1 | 1 | 0 | 0 | 1 | 1 | 0 | 1 | 0 | 1 | 0 | 9 |
| **Kavle et al 2015** | 1 | 1 | 1 | 1 | 1 | 0 | 0 | 1 | 1 | 0 | 1 | 0 | 1 | 0 | 9 |
| **Khatab 2010** | 1 | 1 | 1 | 1 | 1 | 0 | 0 | 1 | 1 | 0 | 1 | 0 | 1 | 0 | 9 |
| **Kiarie et al. 2021** | 1 | 1 | 1 | 1 | 0 | 0 | 0 | 1 | 1 | 0 | 1 | 0 | 1 | 1 | 9 |
| **Musa et al 2014** | 1 | 1 | 1 | 1 | 0 | 0 | 0 | 1 | 1 | 0 | 1 | 0 | 1 | 0 | 8 |
| **Seedhom et al. 2014** | 1 | 1 | 1 | 1 | 0 | 0 | 0 | 1 | 1 | 0 | 1 | 0 | 1 | 0 | 8 |
| **Sharaf et al. 2018** | 1 | 1 | 1 | 1 | 1 | 0 | 0 | 1 | 1 | 0 | 1 | 0 | 1 | 0 | 9 |
| **Sulaiman et al. 2018** | 1 | 1 | 1 | 1 | 1 | 0 | 0 | 1 | 1 | 0 | 1 | 0 | 1 | 0 | 9 |
| **Zotarelli et al. 2007** | 1 | 1 | 1 | 1 | 1 | 0 | 0 | 1 | 1 | 0 | 1 | 0 | 1 | 1 | 10 |

**Table S3.** Determinants of undernutrition (stunting, wasting and underweight) reported by one study.

|  | | **Stunting** | | | | | | | | | **Wasting** | | | | | **Underweight** | | | | | |  |  |  |  |  |  |  |  |  |  |  |
| --- | --- | --- | --- | --- | --- | --- | --- | --- | --- | --- | --- | --- | --- | --- | --- | --- | --- | --- | --- | --- | --- | --- | --- | --- | --- | --- | --- | --- | --- | --- | --- | --- |
|  |  | **El Taguri et al. 2008** | **Elsary etal. 2017** | **Aitsi Selmi 2014** | **Khatab 2010** | **Kiarie et al. 2021** | **Seedhom et al. 2014** | **Sharaf et al. 2018** | **Sulaiman et al. 2018** | **Zottarelli et al. 2007** | **Elsary et al. 2017** | **Khatab 2010** | **Kiarie et al. 2021** | **Sulaiman et al. 2018** | **Zottarelli et al. 2007** | **Elsary et al. 2017** | **Khatab 2010** | **Kiarie et al. 2021** | **Dahab et al. 2020** | **Sulaiman et al. 2018** | **Zottarelli et al. 2007** |  |  |  |  |  |  |  |  |  |  |  |
|  |  | **Libya** | **Egypt** | **Egypt** | **Egypt** | **Sudan** | **Egypt** | **Egypt** | **Sudan** | **Egypt** | **Egypt** | **Egypt** | **Sudan** |  | **Egypt** | **Egypt** | **Egypt** | **Sudan** | **Sudan** |  | **Egypt** |  |  |  |  |  |  |  |  |  |  |  |
| **Rural areas** | **OR** | - | - | - | - | - | - | - | - | - | - | - | - | - | 1.13 | - | - | - | - |  | - |  |  |  |  |  |  |  |  |  |  |  |
|  | **95% CI** | - | - | - | - | - | - | - | - | - | - | - | - | - | 0.87, 1.46 | - | - | - | - |  | - |  |  |  |  |  |  |  |  |  |  |  |
| **Specific geographical locations** | **OR** | 1.67  (Al-Akhdar) | - | - | - | - | - | - | 1.16 (Berber) | - | - | - | - | 1.39 Atbara | - | - | - | - | - | 1.74  Abo Hamad | - |  |  |  |  |  |  |  |  |  |  |  |
|  | **95% CI** | 1.08, 2.58 | - | - | - | - | - | - | 0.9, 1.51 | - | - | - | - | 0.56, 3.44 | - | - | - | - | - | 1.15, 2.64 | - |  |  |  |  |  |  |  |  |  |  |  |
| **Poor household wealth index** | **OR** | - | - | - | - | - | - | - |  | - | - | - | - |  | - | - | - | - | 2.54 | - | - |  |  |  |  |  |  |  |  |  |  |  |
|  | **95% CI** | - | - | - | - | - | - | - |  | - | - | - | - |  | - | - | - | - | 1.52, 4.28 | - | - |  |  |  |  |  |  |  |  |  |  |  |
| **High-intensity conflict level** | **OR** | - | - | - | - | - | - | - |  | - | - | - | - |  | - | - | - | - | 0.94 | - | - |  |  |  |  |  |  |  |  |  |  |  |
|  | **95% CI** | - | - | - | - | - | - | - |  | - | - | - | - |  | - | - | - | - | 1.01, 1.33 | - | - |  |  |  |  |  |  |  |  |  |  |  |
|  |  | **Family and caregiver conditions** | | | | | | | | | | | | | | | | | | | |  |  |  |  |  |  |  |  |  |  |  |
| **Mother working status (no)** | **OR** | - | - | - | - | - | - | - | - | - | - | - | - |  | 0.82 | - | - | - |  | - | 1.27 |  |  |  |  |  |  |  |  |  |  |  |
|  | **95% CI** | - | - | - | - | - | - | - | - | - | - | - | - |  | 0.6, 1.1 | - | - | - |  | - | 0.49, 1.73 |  |  |  |  |  |  |  |  |  |  |  |
| **Maternal height <160** | **OR** | - | - | - | - | - | - | - | - | - | - | - | - |  | 0.82 | - | - | - | - | - | 1.6 |  |  |  |  |  |  |  |  |  |  |  |
|  | **95% CI** | - | - | - | - | - | - | - | - | - | - | - | - |  | 0.63, 1.08 | - | - | - | - | - | 1.25, 1.96 |  |  |  |  |  |  |  |  |  |  |  |
| **Maternal age >35 years** | **OR** | - | - | - | - | - | - | - | - | - | - | - | - |  | 0.86 | - | - | - | - | - | 1.16 |  |  |  |  |  |  |  |  |  |  |  |
|  | **95% CI** | - | - | - | - | - | - | - | - | - | - | - | - |  | 0.56, 1.33 | - | - | - | - | - | 0.85, 1.58 |  |  |  |  |  |  |  |  |  |  |  |
| **Mother pregnant** | **OR** | - | - | - | - | - | - | 0.91 | - | - | - | - | - |  | - | - | - | - | - | - | - |  |  |  |  |  |  |  |  |  |  |  |
|  | **95% CI** | - | - | - | - | - | - | 0.89, 1.07 | - | - | - | - | - |  | - | - | - | - | - | - | - |  |  |  |  |  |  |  |  |  |  |  |
| **Non educated father** | **OR** | - | - | - | - | - | - | - | - | - | - | - | - |  | 0.86 |  | - | - | - | - | 1.67 |  |  |  |  |  |  |  |  |  |  |  |
|  | **95% CI** | - | - | - | - | - | - | - | - | - | - | - | - |  | 0.64, 1.16 |  | - | - | - | - | 1.36, 2.06 |  |  |  |  |  |  |  |  |  |  |  |
| **Consanguinity (first degree)** | **OR** | - | - | - | - | - | - | - | - | - | - | - | - |  | 0.9 | - | - | - | - |  | 1.19 |  |  |  |  |  |  |  |  |  |  |  |
|  | **95% CI** | - | - | - | - | - | - | - | - | - | - | - | - |  | 0.67, 1.21 | - | - | - | - |  | 0.96, 1.48 |  |  |  |  |  |  |  |  |  |  |  |
| **Family size** **9 ±4** | **OR** | - | - | - | - | 1 | - | - | - | - | - | - | 1.05 |  | - | - | - | 1.09 | - |  | - |  |  |  |  |  |  |  |  |  |  |  |
|  | **95% CI** | - | - | - | - | 0.97, 1.06 | - | - | - | - | - | - | 0.94, 1.18 |  | - | - | - | 1.01, 1.18 | - |  | - |  |  |  |  |  |  |  |  |  |  |  |
| **Number of siblings > 5** | **OR** | 1.18 |  | - | - | - | - | - | - | - | - | - | - |  | - | - | - | - | - |  | - |  |  |  |  |  |  |  |  |  |  |  |
|  | **95 % CI** | 1.00, 1.40 |  | - | - | - | - | - | - | - | - | - | - |  | - | - | - | - | - |  | - |  |  |  |  |  |  |  |  |  |  |  |
| **Number of other children < 5 yrs. old in household** | **OR** | - | - | - | - | - | - | 1.12 | - | - | - | - | - |  | - | - | - | - | - |  | - |  |  |  |  |  |  |  |  |  |  |  |
|  | **95% CI** | - | - | - | - | - | - | 1.02, 1.16 | - | - | - | - | - |  | - | - | - | - | - |  | - |  |  |  |  |  |  |  |  |  |  |  |
| **Child a twin** | **OR** | - | - | - | - | - | - | 0.79 | - | - | - | - | - |  | - | - | - | - | - |  | - |  |  |  |  |  |  |  |  |  |  |  |
|  | **95% CI** | - | - | - | - | - | - | 0.58, 0.81 | - | - | - | - | - |  | - | - | - | - | - |  | - |  |  |  |  |  |  |  |  |  |  |  |
| **Birth order of child > 5** | **OR** | - | - | - | - | - | - | - | - | 1.54 |  | - | - |  | 1.13 | - | - | - |  |  | 1.69 |  |  |  |  |  |  |  |  |  |  |  |
|  | **95 % CI** | - | - | - | - | - | - | - | - | 1.37, 1.73 |  | - | - |  | 0.84, 1.54 | - | - | - |  |  | 1.38., 2.08 |  |  |  |  |  |  |  |  |  |  |  |
| **Short birth interval < 23** | **OR** | - | - | - | - | - | - | - | - | - | - | - | - |  | 1.23 | - | - | - |  |  | 1.29 |  |  |  |  |  |  |  |  |  |  |  |
|  | **95 % CI** | - | - | - | - | - | - | - | - | - | - | - | - |  | 0.9, 1.68 | - | - | - |  |  | 1.07, 1.56 |  |  |  |  |  |  |  |  |  |  |  |
| **Non-resident status** | **OR** | - | - | - |  | 0.89 |  | - | - | - | - | - | 4.2 |  | - | - | - | 0.79 | - |  | - |  |  |  |  |  |  |  |  |  |  |  |
|  | **95 % CI** | - | - | - |  | 0.56, 1.43 |  | - | - | - | - | - | 1.44, 12.15 |  | - | - | - | 0.3, 2.11 | - |  | - |  |  |  |  |  |  |  |  |  |  |  |
| **Female is the house head** | **OR** | - | - | - |  | 1.12 | - | - | - | - | - | - | 0.68 |  | - | - | - | 0.44 | - |  | - |  |  |  |  |  |  |  |  |  |  |  |
|  | **95 % CI** | - | - | - |  | 0.71, 1.77 | - | - | - | - | - | - | 0.15, 3.06 |  | - | - | - | 0.13, 1.46 | - |  | - |  |  |  |  |  |  |  |  |  |  |  |
| **Health insurance (yes)** | **OR** | - | - | - | - | - | - | 1.17 | - | - | - | - | - |  | - | - | - | - | - |  | - |  |  |  |  |  |  |  |  |  |  |  |
|  | **95 % CI** | - | - | - | - | - | - | 1.18, 1.3 | - | - | - | - | - |  | - | - | - | - | - |  | - |  |  |  |  |  |  |  |  |  |  |  |
| **family rarely or never play with child** | **OR** | 2.20 |  |  |  |  |  |  | - |  |  |  |  |  |  |  |  |  |  |  |  |  |  |  |  |  |  |  |  |  |  |  |
|  | **95 % CI** | 1.41, 3.42 |  |  |  |  |  |  | - |  |  |  |  |  |  |  |  |  |  |  |  |  |  |  |  |  |  |  |  |  |  |  |
| **Family goes no visits or trip** | **OR** | 1.77 |  |  |  |  |  |  | - |  |  |  |  |  |  |  |  |  |  |  |  |  |  |  |  |  |  |  |  |  |  |  |
|  | **95 % CI** | 1.42,2020 |  |  |  |  |  |  | - |  |  |  |  |  |  |  |  |  |  |  |  |  |  |  |  |  |  |  |  |  |  |  |
| **Agriculture is the main activity (yes)** | **OR** | - | - | - | - | 2.32 | - | - | - | - | - | - | - |  | - | - | - | 1.97 | - |  | - |  |  |  |  |  |  |  |  |  |  |  |
|  | **95% CI** | - | - | - | - | 1.28, 4.21 | - | - | - | - | - | - | - |  | - | - | - | 0.59, 6.60 | - |  | - |  |  |  |  |  |  |  |  |  |  |  |
| **Agriculture is the main activity (No)** | **OR** |  |  |  |  |  |  |  | - |  |  |  | 1.06 |  |  |  |  |  |  |  |  |  |  |  |  |  |  |  |  |  |  |  |
|  | **95% CI** |  |  |  |  |  |  |  | - |  |  |  | 0.77, 1.47 |  |  |  |  |  |  |  |  |  |  |  |  |  |  |  |  |  |  |  |
|  | **Household environmental factors** | | | | | | | | | | | | | | | | | | | | |  |  |  |  |  |  |  |  |  |  |  |
| **No watching TV.** | **OR** | 1.75 | - | - | - | - | - | - |  | - | - | - | - |  | - | - | - | - | - | - |  |  |  |  |  |  |  |  |  |  |  |  |
|  | **95 % CI** | 1.35, 2.26 | - | - | - | - | - | - |  | - | - | - | - |  | - | - | - | - | - | - |  |  |  |  |  |  |  |  |  |  |  |  |
| **Family not listening to radio** | **OR** | 1.17 |  |  |  |  |  |  |  |  |  |  |  |  |  |  |  |  |  |  |  |  |  |  |  |  |  |  |  |  |  |  |
|  | **95 % CI** | 1.01, 1.35 |  |  |  |  |  |  |  |  |  |  |  |  |  |  |  |  |  |  |  |  |  |  |  |  |  |  |  |  |  |  |
| **Electricity** | **OR** | - | - | - | 0.94 | - | - | - |  | - | - | 0.96 |  |  | - | - | 0.93 | - | - | - |  |  |  |  |  |  |  |  |  |  |  |  |
|  | **95% CI** | - | - | - | 0.84, 1.04 | - | - | - |  | - | - | 0.87, 1.04 |  |  | - | - | o.85, 3.27 | - | - | - |  |  |  |  |  |  |  |  |  |  |  |  |
| ***Household environment** | **OR** | 10.15 | - | - | - | - | - | - |  | - | - | - | - |  | - | - | - | - | - | - |  |  |  |  |  |  |  |  |  |  |  |  |
|  | **95% CI** | 2.22, 64 | - | - | - | - | - | - |  | - | - | - | - |  | - | - | - | - | - | - |  |  |  |  |  |  |  |  |  |  |  |  |
| **Had radio** | **OR** | - | - | - | 0.99 | - | - | - |  | - | - | 1.03 | - |  | - | - | 1.02 | - | - | - |  |  |  |  |  |  |  |  |  |  |  |  |
|  | **95% CI** | - | - | - | 0.97, 1.02 | - | - | - |  | - | - | 1.0, 1.05 | - |  | - | - | 1.0, 1.04 | - | - | - |  |  |  |  |  |  |  |  |  |  |  |  |
| **Own flush toilet** | **OR** | - | - | - | 0.97 | - | - | - |  | - | - | 0.95 | - |  | - | - | 1.02 | - | - | - |  |  |  |  |  |  |  |  |  |  |  |  |
|  | **95% CI** | - | - | - | 0.97, 1.02 | - | - | - |  | - | - | 0.88, 1.02 | - |  | - | - | 0.98, 1.06 | - | - | - |  |  |  |  |  |  |  |  |  |  |  |  |
| **Source of water** | **OR** | - | - | - | - | - | - | - |  | - | - | 0.99 | - |  | - | - | 1.1 | - | - | - |  |  |  |  |  |  |  |  |  |  |  |  |
|  | **95% CI** | - | - | - | - | - | - | - |  | - | - | 0.96, 1.02 | - |  | - | - | 0.97, 1.0 | - | - | - |  |  |  |  |  |  |  |  |  |  |  |  |
| **Insecticide nets** | **OR** | - | - | - | - | 1.12 | - | - |  | - | - | - | 0.43 |  | - | - | - | 2.2 | - | - |  |  |  |  |  |  |  |  |  |  |  |  |
|  | **95% CI** | - | - | - | - | 0.74, 1.69 | - | - |  | - | - | - | 0.15, 1.25 |  | - | - | - | 0.82, 5.85 | - | - |  |  |  |  |  |  |  |  |  |  |  |  |
|  | **Health care services** | | | | | | | | | | | | | | | | | | | | |  |  |  |  |  |  |  |  |  |  |  |
| **Incomplete immunisation** | **OR** | 1.28 |  | - | - | - | - | - |  | - | - | - | - |  | - | - | - | - | - | - |  |  |  |  |  |  |  |  |  |  |  |  |
|  | **95% CI** | 1.06, 1.55 |  | - | - | - | - | - |  | - | - | - | - |  | - | - | - | - | - | - |  |  |  |  |  |  |  |  |  |  |  |  |
| **Measles vaccination (yes)** | **OR** | - | - | - | - | 1.65 | - | - |  | - | - | - | 0.70 |  | - | - | - | 1.64 | - | - |  |  |  |  |  |  |  |  |  |  |  |  |
|  | **95% CI** | - | - | - | - | 0.84, 3.25 | - | - |  | - | - | - | 0.15 3.21 |  | - | - | - | 0.38, 7.08 | - | - |  |  |  |  |  |  |  |  |  |  |  |  |
| **Vitamin A supplementation** | **OR** | - | - | - | - | 0.93 |  | - |  | - | - | - | 1.46 |  | - | - | - | 1.6 | - | - |  |  |  |  |  |  |  |  |  |  |  |  |
|  | **95% CI** | - | - | - | - | 0.62, 1.41 |  | - |  | - | - | - | 0.42, 5.29 |  | - | - | - | 0.64, 3.99 | - | - |  |  |  |  |  |  |  |  |  |  |  |  |
| **Place of delivery (home)** | **OR** | 1.65 | - | - | - | - | - | - |  | - | - | - | - |  | - | - | - | - | - | - |  |  |  |  |  |  |  |  |  |  |  |  |
|  | **95% CI** | 1.26, 2.17 | - | - | - | - | - | - |  | - | - | - | - |  | - | - | - | - | - | - |  |  |  |  |  |  |  |  |  |  |  |  |
| **** Mothers’ knowledge on nutrition** | **OR** | - | - | - | - | - | 2.00 | - |  | - | - | - | - |  | - | - | - | - | - | - |  |  |  |  |  |  |  |  |  |  |  |  |
|  | **95% CI** | - | - | - | - | - | 1.47, 2.92 | - |  | - | - | - | - |  | - | - | - | - | - | - |  |  |  |  |  |  |  |  |  |  |  |  |
| **Treatment during pregnancy** | - | - | - | - | - | - | - | - |  | - | - | - | - |  | - | - | - | - | - | - |  |  |  |  |  |  |  |  |  |  |  |  |
|  | - | - | - | - | - | - | - | - |  | - | - | - | - |  | - | - | - | - | - | - |  |  |  |  |  |  |  |  |  |  |  |  |
|  | **Breast feeding** | | | | | | | | | | | | | | | | | | | | |  |  |  |  |  |  |  |  |  |  |  |
| **Child ever breast feed (no)** | **OR** | - | - | 0.89 | - | - | - | - |  | - | - | - | - |  | - | - | - | - | - | - |  |  |  |  |  |  |  |  |  |  |  |  |
|  | **95% CI** | - | - | 0.4, 2.01 | - | - | - | - |  | - | - | - | - |  | - | - | - | - | - | - |  |  |  |  |  |  |  |  |  |  |  |  |
| **Time breast feeding started > 6 hrs.** | **OR** | 0.74 |  | - | - | - | - | - |  | - | - | - | - |  | - | - | - | - | - | - |  |  |  |  |  |  |  |  |  |  |  |  |
|  | **95% CI** | 0.60, 0.92 |  | - | - | - | - | - |  | - | - | - | - |  | - | - | - | - | - | - |  |  |  |  |  |  |  |  |  |  |  |  |
| *****Breast feeding practice inappropriate** | **OR** | 0.77 | - | - | - | - | - | - |  | - | - | - | - |  | - | - | - | - | - | - |  |  |  |  |  |  |  |  |  |  |  |  |
|  | **95% CI** | 0.65, 0.91 | - | - | - | - | - | - |  | - | - | - | - |  | - | - | - | - | - | - |  |  |  |  |  |  |  |  |  |  |  |  |
|  | **Complementary feeding and weaning practices** | | | | | | | | | | | | | | | | | | | | |  |  |  |  |  |  |  |  |  |  |  |
| **Weaning** | **OR** | 1.29 (S) | 1.48 | - | - | - | - | - |  | - | 13.6 (I) | - | - |  | - | - | - | - | - | - |  |  |  |  |  |  |  |  |  |  |  |  |
|  | **95% CI** | 1.08, 1.54 | 0.29, 7.49 | - | - | - | - | - |  | - | 2.70, 68.6 | - | - |  | - | - | - | - | - | - |  |  |  |  |  |  |  |  |  |  |  |  |
| **Frequency of complementary feeding (4-6 times)** | **OR** | - | - | - | - | - | 2.00 | - |  | - | - | - | - |  | - | - | - | - | - | - |  |  |  |  |  |  |  |  |  |  |  |  |
|  | **95% CI** | - | - | - | - | - | 1.52, 2.75 | - |  | - | - | - | - |  | - | - | - | - | - | - |  |  |  |  |  |  |  |  |  |  |  |  |
| **Starting complementary feeding in early ages** | **OR** | - | - | - | - | - | 1.6 | - |  | - | - | - | - |  | - | - | - | - | - | - |  |  |  |  |  |  |  |  |  |  |  |  |
|  | **95% CI** | - | - | - | - | - | 1.05, 2.14 | - |  | - | - | - | - |  | - | - | - | - | - | - |  |  |  |  |  |  |  |  |  |  |  |  |
| **Age when powdered milk given <4 months** | **OR** | 0.84 | - | - | - | - | - | - |  | - | - | - | - |  | - | - | - | - | - | - |  |  |  |  |  |  |  |  |  |  |  |  |
|  | **95% CI** | 0.71, 0.99 | - | - | - | - | - | - |  | - | - | - | - |  | - | - | - | - | - | - |  |  |  |  |  |  |  |  |  |  |  |  |
| **Delayed cow milk introduction> 12 months** | **OR** | 0.69 |  | - |  | - | - | - |  | - | - | - | - |  | - | - | - | - | - | - |  |  |  |  |  |  |  |  |  |  |  |  |
|  | **95 % CI** | 0.53, 0.89 |  | - |  | - | - | - |  | - | - | - | - |  | - | - | - | - | - | - |  |  |  |  |  |  |  |  |  |  |  |  |
|  | **Dietary intake** | | | | | | | | | | | | | | | | | | | | |  |  |  |  |  |  |  |  |  |  | - |
| ****** Poor food consumption profile** | **OR** | - | - |  |  | - | - | - |  | - | - | - | - |  | - | - | - | - | 0.93 | - | |  |  |  |  |  |  |  |  |  |  |  |
|  | **95% CI** | - | - |  |  | - | - | - |  | - | - | - | - |  | - | - | - | - | 0.64, 1.37 | - |  |  |  |  |  |  |  |  |  |  |  |  |
| **Bottle feed** | **OR** | 0.74 | - | - | - | - | - | - |  | - | - | - | - |  | - | - | - | - | - | - |  |  |  |  |  |  |  |  |  |  |  |  |
|  | **95% CI** | 0.64, 0.87 | - | - | - | - | - | - |  | - | - | - | - |  | - | - | - | - | - | - |  |  |  |  |  |  |  |  |  |  |  |  |
| **Dietary diversity** | **OR** | 0.81 | - | - |  | - | - | - |  | - | - | - | - |  | - | - | - | - | - | - |  |  |  |  |  |  |  |  |  |  |  |  |
|  | **95 % CI** | 0.7, 0.92 | - | - |  | - | - | - |  | - | - | - | - |  | - | - | - | - | - | - |  |  |  |  |  |  |  |  |  |  |  |  |
| **Child given fruit/ vegetables in the last 24 hrs** | **OR** | - | - | 0.87 | - | - | - | - |  | - | - | - | - |  | - | - | - | - | - | - |  |  |  |  |  |  |  |  |  |  |  |  |
|  | **95 % CI** | - | - | 0.64, 1.19 | - | - | - | - |  | - | - | - | - |  | - | - | - | - | - | - |  |  |  |  |  |  |  |  |  |  |  |  |
| **Given sugary snack in the last 24 hrs** | **OR** | - | - | 0.78 | - | - | - | - |  | - | - | - | - |  | - | - | - | - | - | - |  |  |  |  |  |  |  |  |  |  |  |  |
|  | **95 % CI** | - | - | 0.59, 1.05 | - | - | - | - |  | - | - | - | - |  | - | - | - | - | - | - |  |  |  |  |  |  |  |  |  |  |  |  |
|  |  | **Child health** | | | | | | | | | | | | | | | | | | | |  |  |  |  |  |  |  |  |  |  |  |
| **Presence of diarrhoea** | **OR** | - | - | - | - | - | - | - |  | - | - | - | - |  | - | - | - | - | 1.54 | - |  |  |  |  |  |  |  |  |  |  |  |  |
|  | **95% CI** | - | - | - | - | - | - | - |  | - | - | - | - |  | - | - | - | - | 1.31, 1.8 | - |  |  |  |  |  |  |  |  |  |  |  |  |
| **Gastroenteritis** | **OR** | - | 4.82 |  | - | - | - | - |  | - | 7.89 | - | - |  | - | 11.22 | - | - | - | - |  |  |  |  |  |  |  |  |  |  |  |  |
|  | **95% CI** | - | 1.75, 13.3 |  | - | - | - | - |  | - | 2.77, 22.44 | - | - |  | - | 3.35, 35.72 | - | - | - | - |  |  |  |  |  |  |  |  |  |  |  |  |
| **Parasitic infestation** | **OR** | - | 3.3 | - | - | - | - | - |  | - | 11.72 | - | - |  | - | 22.2 | - | - | - | - |  |  |  |  |  |  |  |  |  |  |  |  |
|  | **95% CI** | - | 1.86, 5.83 | - | - | - | - | - |  | - | 6.53, 21.04 | - | - |  | - | 11.85, 41.72 | - | - | - | - |  |  |  |  |  |  |  |  |  |  |  |  |
| **2 weeks retrospective morbidity** | **OR** | - | - | - | - | 1.13 | - | - |  | - | - | - | 1.59 |  | - | - | - | 2.02 | - | - |  |  |  |  |  |  |  |  |  |  |  |  |
|  | **95% CI** | - | - | - | - | 0.77, 1.66 | - | - |  | - | - | - | 0.53, 4.81 |  | - | - | - | 0.91, 4.52 | - | - |  |  |  |  |  |  |  |  |  |  |  |  |
| **Dewormed in the last three months** | **OR** | - | - | - | - | 1.08 | - | - |  | - | - | - | 0.66 |  | - | - | - | 1.4 | - | - |  |  |  |  |  |  |  |  |  |  |  |  |
|  | **95% CI** | - | - | - | - | 0.73, 1.61 | - | - |  | - | - | - | 0.19, 2.28 |  | - | - | - | 0.60, 0.32 | - | - |  |  |  |  |  |  |  |  |  |  |  |  |

***Household environment:** dwelling type, kitchen location, water storage, water treatment, garbage disposal metho**d**

**** Mother’s knowledge on nutrition:** The nutritional knowledge scores for all mothers were divided into low=3-6, moderate=7-11, and high=12-16

*****Inappropriate breastfeeding:** breast-feeding was considered optimal if it started early if it was continued for 12 months and if weaning started at 6–8 months.

**(S) =** Sudden weaning

**(I) =** Improper weaning

******Poor food consumption profile:** Based on a weighted food consumption method, the score was calculated using the World Food Programme's Analysis and Mapping (VAM) seven-day recall method.

*******Dietary diversity:** given pasteurized bottled milk, given powdered milk, preserved baby food and juice
